# ISG15 induces ESRP1 to inhibit lung adenocarcinoma progression

**DOI:** 10.1038/s41419-020-2706-7

**Published:** 2020-07-02

**Authors:** Tongyuan Qu, Wenshuai Zhang, Lisha Qi, Lu Cao, Changxu Liu, Qiujuan Huang, Guangning Li, Lingmei Li, Yalei Wang, Qianru Guo, Yuhong Guo, Danyang Ren, Yanan Gao, Jinpeng Wang, Bin Meng, Bin Zhang, Wenfeng Cao

**Affiliations:** 1 Department of Pathology, Tianjin Medical University Cancer Institute and Hospital, National Clinical Research Center for Cancer, Key Laboratory of Cancer Prevention and Therapy, Tianjin’s Clinical Research Center for Cancer, Tianjin Medical University, 300060 Tianjin, China; 2 Department of Pathology, Tianjin Academy of Traditional Chinese Medicine Affiliated Hospital, 300120 Tianjin, China; 3 Department of Breast Cancer, Tianjin Medical University Cancer Institute and Hospital, National Clinical Research Center for Cancer, Key Laboratory of Breast Cancer Prevention and Therapy, Tianjin’s Clinical Research Center for Cancer, Tianjin Medical University, 300060 Tianjin, China

**Keywords:** Transcriptional regulatory elements, Cancer prevention, Non-small-cell lung cancer, Tumour-suppressor proteins, Translational research

## Abstract

Our previous work demonstrated that Epithelial Splicing Regulatory Protein 1 (ESRP1) could inhibit the progression of lung adenocarcinoma (ADC). When ESRP1 was upregulated, the interferon (IFN) pathway was activated and Interferon-stimulated gene 15 (ISG15) expression increased exponentially in our microarray result. In this study, we aim to explore the function of ISG15 and its interactions with ESRP1 and to provide new insights for ADC treatment. ISG15 expression in lung ADC tissues was determined by immunohistochemistry (IHC) staining. The effect of ISG15 on lung ADC progression was examined by in vitro and in vivo assays. The mechanism of action on ESRP1 regulating ISG15 was investigated using Western blotting, RT-qPCR, immunofluorescence staining, chromatin immunoprecipitation, and a dual luciferase reporter system. The ISGylation between ISG15 and ESRP1 was detected by co-immunoprecipitation. Patients with high ISG15 expression were associated with higher survival rates, especially those with ISG15 expression in the nucleus. In vitro and in vivo experiments showed that upregulation of ISG15 inhibited EMT in lung ADC. ESRP1 upregulated the expression of ISG15 through CREB with enriched ISG15 in the nucleus. Importantly, ISG15 promoted ISGylation of ESRP1 and slowed the degradation of ESRP1, which demonstrated that ESRP1 and ISG15 formed a positive feedback loop and jointly suppressed EMT of lung ADC. In conclusion, ISG15 serves as an independent prognostic marker for long-term survival in lung ADC patients. We have revealed the protective effect of ISG15 against lung ADC progression and the combinatorial benefit of ISG15 and ESRP1 on inhibiting EMT. These findings suggest that reconstituting ISG15 and ESRP1 may have the potential for treating lung ADC.

## Introduction

Lung cancer is one of the most common malignancies worldwide, and ~85% of lung cancers are NSCLCs^1^. Lung ADC is the main type of NSCLC, accounting for ~60% of all NSCLCs^2,3^. Unfortunately, although drugs that target tyrosine kinase receptors have been developed in recent years, most NSCLC patients still have tumour progression^4^. Therefore, it is especially important to explore new targets that can inhibit the malignant behaviour of lung ADC.

Epithelial Splicing Regulatory Protein 1 (ESRP1), also known as RBM35A, is a member of the RNA-binding protein FOX2 homologue family^5^ and is also a key molecule involved in regulating the alternative splicing process, which plays an important role in epithelial–mesenchymal transition (EMT)^6,7^. Our previous results have shown that ESRP1 is an independent prognostic factor for lung ADC. ESRP1 downregulation is closely related to advanced TNM stage, metastasis and lymph node metastasis. ESRP1 inhibits EMT in lung ADC by participating in the alteration of the CD44 subtype and FGFR2 subtype.

Based on this background, we performed microarray gene expression in ESRP1-overexpressing lung ADC cells (A549) to detect dozens of significantly altered pathways and genes, and the IFN pathway was most significantly altered. ISG15 is a type I interferon-inducible gene that encodes a protein with pleiotropic function and can be used as both a soluble molecule and a protein modifier. ISG15, like the signalling factors STAT1 and STAT2^8^, is a key downstream component of the IFN pathway^9,10^. As the first discovered ubiquitin-like protein, ISG15 contains two Ubl domains joined by small regions, with 33% and 32% homology to ubiquitin proteins^11^. ISG15 can be modified at the post-translational level by conjugating its C-terminal glycine residue to the side chain amino group of the target protein lysine residue, which is called ISGylation^11,12^. ISGylation is very similar to ubiquitination: both can stabilize the protein and affect the activity of the target protein^13^.

ISG15 and ISGylation have been shown to be closely related to cancer^14^. Currently, a large number of studies have shown that ISG15 exerts an anticancer effect^15–17^. Studies have also shown that treatment with exogenous ISG15 protein can reduce the burden of primary and metastatic breast cancer (BC) in mice^18^.

Based on previous articles, this article aims to clarify the role of ISG15 in lung ADC and the relationship between ESRP1 and ISG15. In this paper, ISG15 IHC staining will be used to study its clinicopathological significance in lung ADC tissues. The effects of ISG15 on lung ADC cells in vitro and in vivo are determined by cell experiments and a xenograft mouse model. We will also elucidate the effect of ESRP1 on the expression and localization of ISG15 at the molecular level and the post-translational modification of ESRP1 by ISG15. Therefore, research on ISG15 and ESRP1 in lung ADC may provide new therapeutic targets for the diagnosis, treatment and prognosis of patients with this disease.

## Materials and methods

### Cell culture conditions and treatments

The A549 and H1299 lung ADC cell lines included in the study were obtained from the Type Culture Collection of the Chinese Academy of Sciences, in Shanghai, China.

The cells had been authenticated for STR profiling and tested for mycoplasma by the vendor. Cell culture was performed as described previously^19^. Cells in the logarithmic growth phase were used in the experiments.

### Clinical samples

From January 2012 to December 2012, a total of 153 lung ADC tissue samples were collected from the Department of Pathology at Tianjin Medical University Cancer Institute and Hospital. From 2016 to 2018 at the same place, four paired tissues of distant metastasis/recurrence were collected. All resources were characterized and included patients’ clinical, prognostic and pathological data. None of the patients had received chemotherapy or radiotherapy before operation. The diagnosis of lung ADC was histopathologically confirmed. The protocols in this study were approved by the hospital’s Protection of Human Subjects Committee.

### Immunohistochemistry and haematoxylin and eosin staining

Haematoxylin and eosin (H&E) staining and IHC staining were performed as described previously^19^. The primary antibody information is as follows: anti-ESRP1 antibody (Abcam, ab107278, 1:100) and anti-ISG15 antibody (Santa Cruz Biotechnology, sc-166755, 1:200). Three pathologists (Wengfeng Cao, Lisha Qi and Bin Meng) independently evaluated the expression of ISG15 without understanding the patient’s clinical parameters. The percent positivity was scored as “1” (0%-25%), “2” (26%-50%), “3” (50%-75%), or “4” (>75%). The staining intensity was scored as “0” ((negatively stained) “1” (weakly stained), “2” (moderately stained), or “3” (strongly stained). The multiplier of the positive percentage and staining intensity of the stained area as a result of the total immunostaining score ranged from 0 to 12. A total score of 0-4 indicated no expression or low expression of protein, and a total score of ≥6 indicated high expression. High ISG15 expression was defined as an ISG15 IHC staining score of ≥6 in lung ADC tissues. ISG15 nuclear positivity was defined as the expression of ISG15 IHC staining in the nucleus in lung ADC.

### Western blotting analysis

Western blotting was performed as described previously^19^. The primary antibody information is as follows: anti-ESRP1 antibody (Abcam, ab107278, 1:200), anti-STAT1 antibody (Abcam, ab92056, 1:500), anti-STAT2 antibody (Abcam, ab32367, 1:5000), anti-ISG15 antibody (Santa Cruz Biotechnology, sc-166755, 1:500), anti-CREB antibody (Abcam, ab32515, 1:1000), anti-E-cadherin antibody (Cell Signaling Technology, #14472, 1:500), anti-N-cadherin antibody (Cell Signaling Technology, #13116, 1:500), anti-Vimentin antibody (Abcam, ab8069, 1:500) and anti-GAPDH antibody (Cell Signaling Technology, #3700s, 1:5000).

### Lentiviral infection and plasmid transfection

ESRP1 overexpression, ESRP1 knockdown, ISG15 overexpression and ISG15 knockdown lentivirus were purchased from Genechem (Shanghai, China). Lentiviral infections were performed according to the manufacturer’s protocol. Infected cells were screened with 5 µg/ml puromycin. The expression of ESRP1 and ISG15 was confirmed by Western blotting and RT-qPCR. The CREB plasmid was cloned into a pcDNA3.1 vector (Life Technology). Transfection was performed using Fugene HD transfection reagent (Roche, Indianapolis, IN) according to the manufacturer’s protocol. More details on Sh-ESRP1 and Sh-ISG15 are provided in Table S1.

### Quantitative reverse transcription-PCR

Total RNA was extracted using TRIzol (Invitrogen, USA) from two lung ADC cell lines according to the manufacturer’s protocol. Quantitative reverse transcription-PCR (RT-qPCR) was performed as described previously^20^. Primers for qPCR are summarized in Table S1.

### Immunofluorescence staining

Immunofluorescence staining was performed as described previously^20^. For this, A549 and H1299 cells were stained with anti-ISG15 antibody (Santa Cruz Biotechnology, sc-166755, 1:200), anti-E-cadherin antibody (Cell Signaling Technology, #14472, 1:200), anti-Vimentin antibody (Abcam, ab8069, 1:200) and DAPI (Solarbio, C0065).

### Colony formation assay and CCK-8 cell proliferation assay

Approximately 1000 lung ADC cells were inoculated into each well of a six-well plate and cultured in a 37 °C incubator with a 5% CO2 atmosphere for 10 days. The culture solution was discarded, and cells were fixed with paraformaldehyde for 30 minutes and stained with 0.1% crystal violet for 30 min. Quantification was performed using five independent fields.

Cell proliferation was assessed using Cell Counting Kit-8 (Dojindo, Japan). Lung ADC cells (3 × 10^4^) growing in the log phase were counted and seeded in a 96-well plate in a volume of 200 μl, with five total replicate wells. After inoculation for 24, 48, 72 and 96 h, 20 μl of the reagent was added and incubated for 2 h, and then, the absorbance was measured at 450 nm.

### Transwell and wound healing assay

A transwell assay and wound healing assay were performed as described previously^19^. Quantification was performed using 5 independent fields.

### Xenograft mouse model

Five-week-old BALB/c nude female mice (Si Bei Fu Biotechnology, Beijing, China) were randomly divided into three groups of five. Each nude mouse was injected subcutaneously with 3 × 10^6^ H1299 cells grown in log phase. In the Sh-ISG15 group, ISG15 was knocked down in the cells injected the in nude mice. In the Exo-ISG15 group, nude mice were injected with recombinantly purified ISG15 (1 mg/kg) (R&D Systems, USA) three times a week, from the 3rd week to the 8th week after the cells were injected. According to the formula tumour volume = (width^2^ × length)/2, the length and width of the mouse tumours were observed three times a week. After 8th weeks, the mice were sacrificed to remove the tumours, liver, lungs and other tissues. All in vivo experiments were reviewed and approved by the International Animal Care and Use Committee of Tianjin Medical University Cancer Institute and Hospital.

### Chromatin immunoprecipitation assay

A chromatin immunoprecipitation (ChIP) assay (Millipore, 17-10461) was performed according to the manufacturer’s instructions. ChIP was performed as described previously^19^.

### Dual-luciferase reporter system assay

Assays were performed using a dual-luciferase reporter system kit (Promega). Plasmids (Gengchem, Shanghai, China) containing the ISG15 promoter region (wild type and mutant) and CREB were cotransfected in 293T cells. After 48 h of cell culture, the two luminescence values were determined according to the manufacturer’s protocol, and the relative luminescence values were calculated. Luciferase activity values were determined and normalized to the corresponding Renilla luciferase activity values.

### Co-immunoprecipitation

Co-immunoprecipitation (Co-IP) assays were performed using cellular protein lysates and Pierce Co-IP kits (Thermo Scientific) according to the manufacturer’s protocol. After lysing the cells, 10 µg of anti-ISG15 antibody was added and incubated overnight at 4 °C. The protein complexes were then eluted and detected with an anti-FLAG antibody.

### Statistical analysis

Data are expressed as the mean ± standard deviation (SD) of at least three independent experiments. The sample sizes for relevant experiment were determined by power analysis. When the variance between the two groups was similar, student’s *t* test (two-tailed, paired) was used to analysis data difference between two groups, if not the same, welch’s *t*-test was used, and chi-squared test was used to analysis clinical data. The log-rank test was used to analyse the differences in survival time between the groups. A Cox proportional hazard regression model was used to assess the risk factors related to the prognosis of patients. Data analysis mainly used the software SPSS 23.0 (IBM, USA). Values of *P* < 0.05 (*), *P* < 0.01 (**) and *P* < 0.001 (***) were considered significant.

## Results

### High expression of ISG15, especially in the nucleus, is associated with better prognosis in lung ADC

**Fig. 1:**
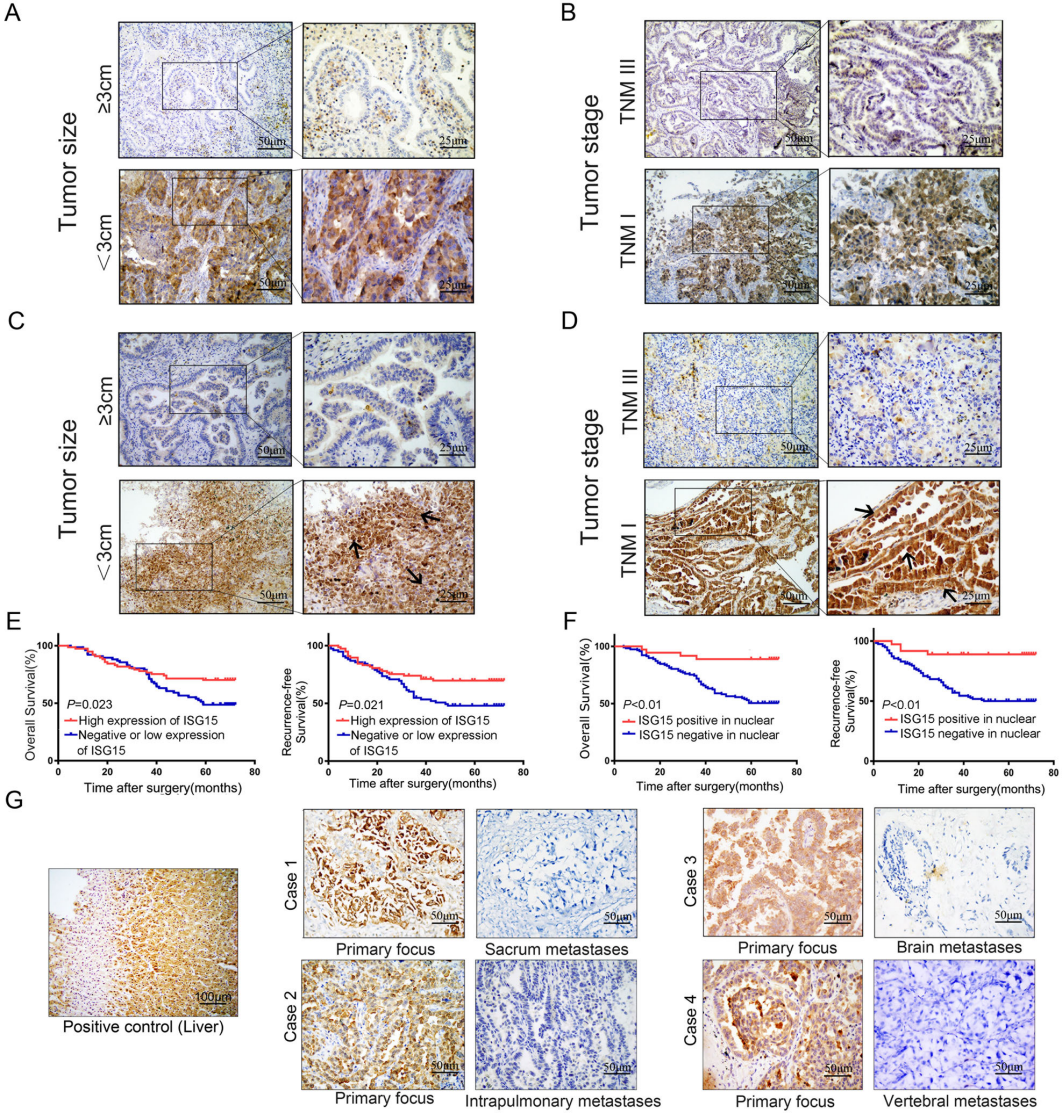
Expression of ISG15 in lung adenocarcinoma and its effect on prognosis. **a**, **b** Representative IHC images showing the expression of ISG15 in different tumour sizes and tumour stages. Magnification, ×200, ×400. **c**, **d** Representative images of ISG15 nuclear expression in different tumour sizes and different tumour stages. The black arrow indicates the presence of ISG15-positive nuclei. Magnification, ×200, ×400. **e** Kaplan–Meier curves showing the OS (left) or RFS (right) of patients with high ISG15 expression vs. negative or low ISG15 expression. **f** Kaplan–Meier curves showing the OS (left) or RFS (right) of patients with ISG15-positive nuclear staining vs. ISG15-negative nuclear staining. **g** The expression of ISG15 in primary and metastatic lesions of four cases was observed by IHC staining. Liver tissue as a positive control. Magnification, ×400, ×800.

First, the expression level of ISG15 in 153 lung ADC tissues was detected by IHC. The results are shown in Table 1. We defined cases with an immune score of ≥6 as ISG15 high expression, and conversely as low or no expression. There were 78 (51.0%) cases with high ISG15 expression, and 75 (49.0%) cases had low or negative ISG15 expression. ISG15 expression was associated with tumour size (*P* < 0.001), pTNM stage (*P* < 0.001), and lymphatic metastasis (*P* = 0.038). ISG15 staining is shown in Fig. 1a, b. We also noticed that some tissues had positive nuclear staining of ISG15. ISG15 is expressed in the nucleus and is defined as ISG15 nuclear positive. In response to this phenomenon, we conducted an analysis, and the results are shown in Table 2. ISG15 nuclear staining was closely related to tumour size (*P* = 0.021), pTNM stage (*P* = 0.011) and lymphatic metastasis (*P* = 0.025) (Fig. 1c, d). Kaplan–Meier survival analysis demonstrated that patients with high expression of ISG15 had better OS (*P* = 0.023) and RFS (*P* = 0.021) (Fig. 1e) than patients with low or negative expression of ISG15, especially those with ISG15 positive nuclear staining who had the best prognosis (*P* < 0.001 for OS, *P* < 0.001 for RFS) (Fig. 1f). Univariate and multivariate prognostic analyses also showed that ISG15 was an independent prognostic factor in lung ADC (Table 3). These data suggest that ISG15 may be a protective factor in lung ADC.

**Table 1:** Correlation between ISG15 and clinicopathologic characteristics of lung adenocarcinoma.

		ISG15 Expression			
Variable	Total	Low or negative (%)	High (%)	*χ*^2^	*P* value
*Age*					
<50	32	17 (53.1)	15 (46.9)	0.273	0.692
≥50	121	58 (47.9)	63 (52.1)		
*Sex*					
Male	66	29 (43.9)	37 (56.1)	1.199	0.328
Female	87	46 (52.9)	41 (47.1)		
*Tumour size (cm)*					
<3	84	30 (35.7)	54 (64.3)	13.195	<0.001*
≥3	69	45 (65.2)	24 (34.8)		
*Histological type*					
Lepidic predominant	24	8 (33.3)	16 (66.7)		
Acinar predominant	62	32 (51.6)	30 (48.4)		
Papillary predominant	12	9 (75.0)	3 (25.0)		
Micropapillary predominant	24	10 (41.7)	14 (58.3)	6.374	0.173
Solid predominant	31	16 (51.6)	15 (48.4)		
*pTNM stages*					
TNMI	105	40 (38.1)	65 (61.9)	16.025	<0.001*
TNMII	32	23 (71.9)	9 (28.1)		
TNMIII	16	12 (75.0)	4 (25.0)		
*Lymph metastasis*					
Absent	103	44 (42.7)	59 (57.3)	5.008	0.038*
Present	50	31 (62.0)	19 (38.0)		

*P-values in bold are statistically significant.

**Table 2:** Correlation between ISG15 positive in nuclear and clinicopathologic characteristics of lung adenocarcinoma.

		ISG15 Nuclear			
Variable	Total	Negative (%)	Positive (%)	*χ*^2^	*P* value
*Age*					
<50	32	26 (81.2)	6 (18.8)	0.514	0.640
≥50	121	91 (75.2)	30 (24.8)		
*Sex*					
Male	66	47 (71.2)	19 (28.8)	1.784	0.248
Female	87	70 (80.5)	17 (19.5)		
*Tumour size(cm)*					
<3	84	58 (69.0)	26 (31.0)	5.704	0.021*
≥3	69	59 (85.5)	10 (14.5)		
*Histological type*					
Lepidic predominant	24	18 (75.0)	6 (25.0)		
Acinar predominant	62	48 (77.4)	14 (22.6)		
Papillary predominant	12	10 (83.3)	2 (16.7)		
Micropapillary predominant	24	19 (79.2)	5 (20.8)	0.993	0.911
Solid predominant	31	22 (71.0)	9 (29.0)		
*pTNM stages*					
TNMI	105	73 (69.5)	32 (30.5)	9.034	0.011*
TNMII	32	29 (90.6)	3 (9.4)		
TNMIII	16	15 (93.8)	1 (6.3)		
*Lymph Metastasis*					
Absent	103	73 (70.9)	30 (29.1)	5.487	0.025*
Present	50	44 (88.0)	6 (12.0)		

*P-values in bold are statistically significant.

**Table 3:** Multivariate Cox proportional hazard regression analysis of OS in lung adenocarcinoma.

	Univariate analysis		Multivariate analysis	
Variable	HR(95% CI)	*P*	HR(95% CI)	*P*
Age, <50 versus ≥50 years	0.992 (0.551–1.786)	0.979	0.652 (0.348–1.222)	0.182
Sex, male versus female	0.870 (0.540–1.402)	0.567	0.727 (0.441–1.198)	0.211
Tumour size, <3 versus ≥ 3 cm	2.762 (1.675–4.553)	**<0.001**	1.462 (0.832–2.568)	0.186
Lymph metastasis, present versus absent	3.380 (2.090–5.465)	<0.001*	1.415 (0.845–2.369)	0.187
Recurrence, present versus absent	17.093 (7.754–37.678)	<0.001*	14.443 (6.232–33.473)	<0.001*
ISG15 expression, high versus low	0.574 (0.353–0.934)	0.025*	0.555 (0.313–0.983)	0.043*
ISG15 positive in nuclear versus negative	0.245 (0.106–0.566)	0.001*	0.352 (0.138–0.896)	0.029*

*P-values in bold are statistically significant.

There were 75 patients with low or no ISG15 expression and up to 47 (62.7%) patients with long-term metastasis and recurrence. Additionally, there were 78 people with high expression of ISG15, of which only 28 (35.9%) had long-term metastasis and recurrence. The result showed that patients with high expression of ISG15 have a lower risk of long-term metastasis and recurrence. Of the 36 patients with ISG15 nuclear positivity, only 8 (22.2%) had long-term recurrence and metastasis. Of the 117 patients without ISG15-positive nuclei, 67 (57.3%) had long-term recurrence and metastasis. Among those patients, the long-term metastasis and recurrence rate of the patients with ISG15-positive nuclei (22.2%) was lower than that of patients with a high expression of ISG15 (35.9%) (Table 4). These results suggest that ISG15-positive nuclei may be a more important prognostic factor in lung ADC.

**Table 4:** Correlation between expression of ISG15/ISG15 in nuclear and recurrence/metastasis of lung ADC.

		Recurrence/metastasis			
Variable	Total	Absent (%)	Present (%)	*χ*^2^	*P* value
*Expression of ISG15*					
Low or negative	75	28 (35.9)	47 (62.7)	10.964	0.001*
High	78	50 (64.1)	28 (35.9)		
*ISG15 nuclear*					
Negative	117	50 (64.1)	67 (89.3)	13.528	<0.001*
Positive	36	28 (35.9)	8 (10.7)		

*P-values in bold are statistically significant.

In addition, we explored changes in ISG15 expression in patients with primary and matched long-term metastases. We selected the patients with the acinar predominant pathological type, pTNM I stage, and absent lymph metastasis at the time of surgery. There were 2 cases with low or negative ISG15 expression and 6 cases with high ISG15 expression. Of these six patients with high ISG15 expression, we obtained specimens of metastatic foci from four cases and stained for ISG15. We were surprised to find that the patients with recurrence/metastases had negative ISG15 staining, which was unlike their primary foci (Fig. 1g). We suspect that the loss of ISG15 led to long-term recurrence and metastasis in these patients. This result further suggests that ISG15 may play an important role in inhibiting the lung ADC process.

### ISG15 inhibits lung adenocarcinoma progression in vitro and in vivo through EMT

We further investigated the effect of ISG15 in lung ADC both in vivo and in vitro.

**Fig. 2:**
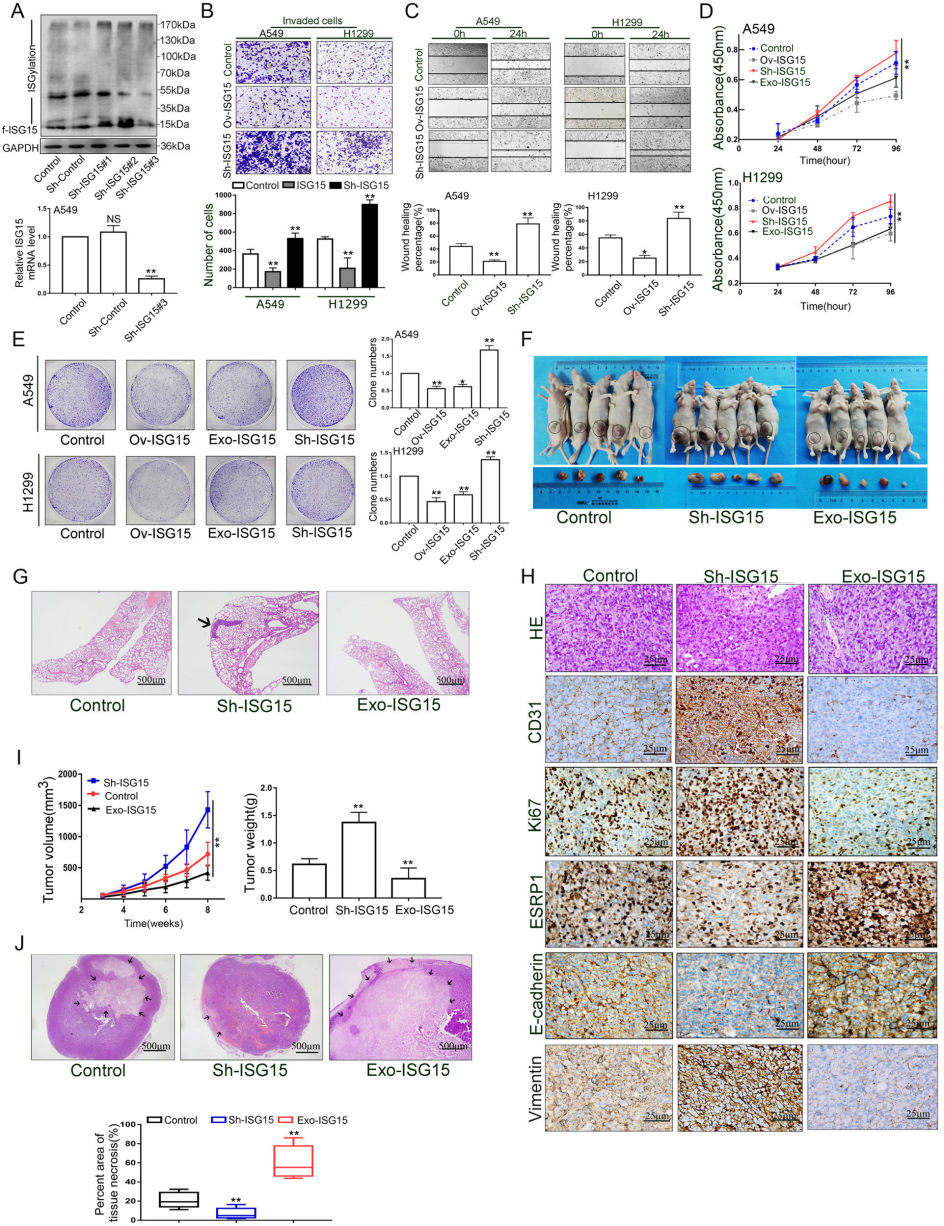
ISG15 inhibits lung adenocarcinoma progression in vitro and in vivo through EMT. **a** Western blotting and RT-qPCR were performed to detect ISG15 expression in A549 cells infected with three independent ISG15-targeted lentiviruses. The data are shown as the mean ± SD. *P < 0.05, **P < 0.01. **b** Transwell assays were used to analyze the invasive ability of ISG15 and Sh-ISG15 A549 and H1299 cells. Data are shown as the mean ± SD. *P < 0.05, **P < 0.01. Magnification, ×200. **c** The effects of ISG15 and Sh-ISG15 on the migration ability of A549 and H1299 cells were analyzed by wound healing assay. Data are shown as the mean ± SD. *P < 0.05, **P < 0.01. Magnification, ×100. **d**, **e** The effects of ISG15, Sh-ISG15 and exogenous ISG15 on the proliferative capacity of A549 and H1299 cells were analyzed by colony formation and CCK-8 assays. The data are shown as the mean ± SD. *P < 0.05, **P < 0.01. **f** Tumour representative images of subcutaneously formed tumours removed from nude mice. **g** HE staining of the lungs remove from the mice in three groups. Black arrows represent images of metastatic nodules in the lungs. Magnification, ×40. **h** Typical HE images of tumours formed subcutaneously in three groups of nude mice. Typical images of IHC staining of CD31, Ki-67, ESRP1, E-cadherin and Vimentin in three groups of subcutaneous tumours in mice. Magnification, ×400. **i** Effects of three treatments on tumour volume (left) and weight (right) formation in nude mice. The data are shown as the mean ± SD. *P < 0.05, **P < 0.01. **j** Typical images (top) and comparisons (bottom) of necrotic areas of tumours formed subcutaneously in three groups of nude mice. The data are shown as the mean ± SD. *P < 0.05, **P < 0.01, Magnification, ×40.

As shown in Fig. 2a, Sh-ISG15#3 had the highest knockdown efficiency. We selected this construct to generate A549 and H1299 cells with stable ISG15 expression (Sh-ISG15). The results of the transwell assay and the wound healting demonstrated that the upregulation of ISG15 suppressed the migration and invasion abilities of these cells, and knockdown of ISG15 promoted these abilities (Fig. 2b, c). Because ISG15 is a soluble molecule, in the CCK-8 analysis and colony formation analysis, we added an experimental group: the exogenous ISG15 (Exo-ISG15) group. The CCK-8 assay and colony-forming assay results demonstrated that the proliferation ability of the Ov-ISG15 group was the lowest compared to the control group. The proliferation ability of the Exo-ISG15 group was slightly higher than that of the Ov-ISG15 group but still much lower than that of the control group (Fig. 2d, e). Overall, the in vitro experiments demonstrated that ISG15 can inhibit the invasion, migration and proliferation abilities of lung ADC cells.

**Fig. 3:**
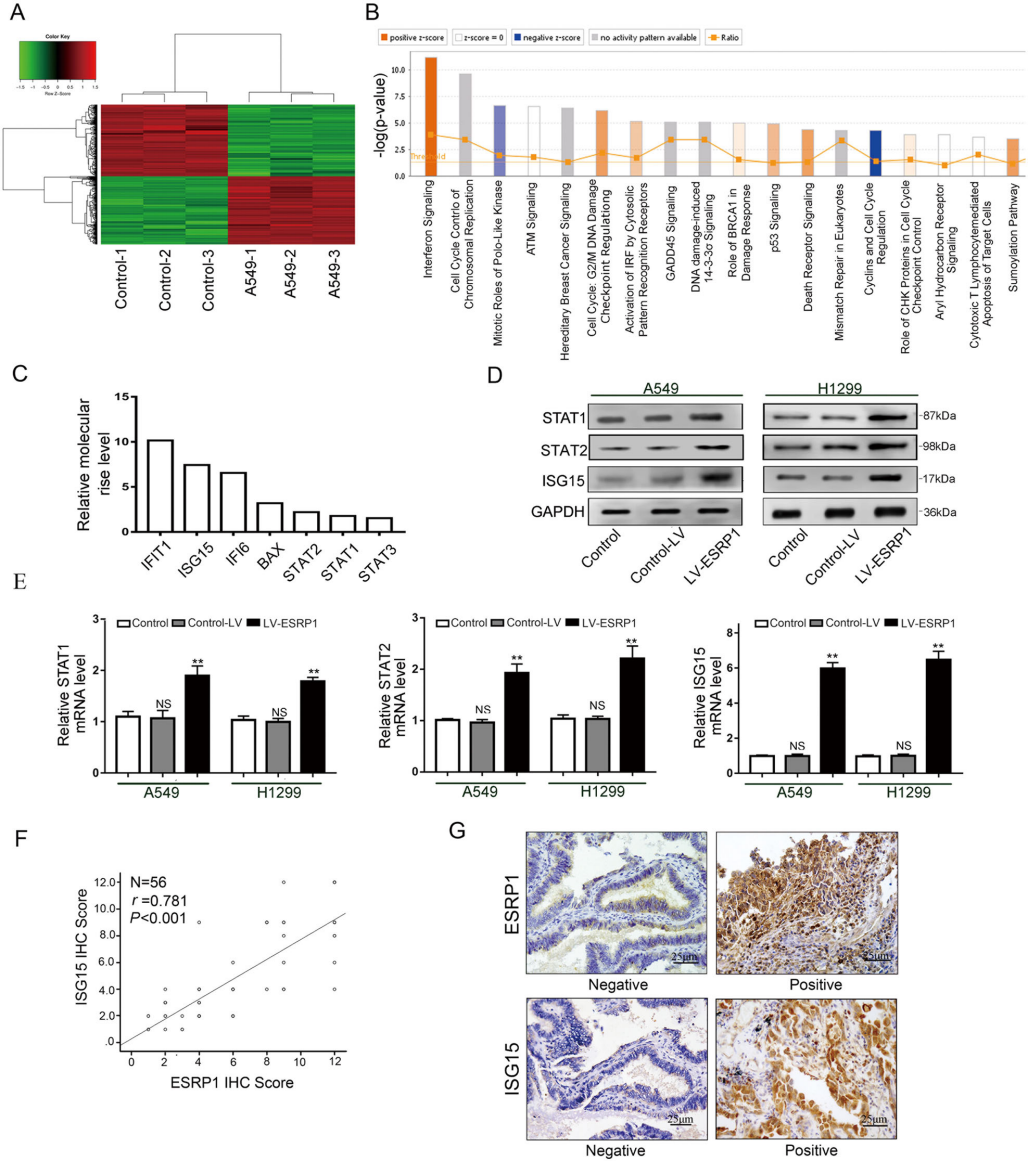
Activation of the IFN pathway and upregulation of ISG15 expression are closely correlated with ESRP1. **a** Microarray data indicates the difference in gene expression between three samples of wild-type A549 cells and three samples of ESRP1-overexpressed cells. **b** All signaling pathways are sorted by −Log (*p*-value). In the figure, the signaling pathways marked in orange represent z-score >0, while the signaling pathways marked in blue represent z-score <0, and z-score >2 represents significant activation of the pathway. Interferon signaling was significantly activated, and the z-score was 3.464. **c** Protein changes in A549 cells when ESRP1 was overexpressed. **d**, **e** When ESRP1 was overexpressed, the changes in STAT1, STAT2 and ISG15 in A549 and H1299 cell lines were detected by RT-qPCR and Western blotting. The data are shown as the mean ± SD. *P < 0.05, **P < 0.01. **f** Expression of ISG15 and ESRP1 in the same lung ADC case. Magnification, ×400. **g** Correlation between ESRP1 IHC score and ISG15 IHC score in 56 patient lung ADC tissues.

Subsequently, we evaluated the effect of ISG15 in vivo using a xenograft mouse model (Fig. 2f). In the Sh-ISG15 group, tumours had the fastest growth rate and had the largest weight compared to the control group (Fig. 2i). In this group, we also found three cases of pulmonary metastasis (Fig. 2g). In contrast, the Exo-ISG15 group had the smallest volume and weight. Quantitative analysis of the tumour necrosis area showed that the Exo-ISG15 group had the largest area (62.1%) and that the Sh-ISG15 group had the smallest area (6.3%) (Fig. 3j). The tumours of these three groups were separately subjected to IHC staining and the expression levels of CD31 (angiogenesis-related indicators), Ki-67, ESRP1 and E-cadherin were observed (Fig. 2h). In the Sh-ISG15 group, the positive density of CD31, Ki-67 and vimentin staining was greater than that in the other two groups, while the positive levels of ESRP1 and E-cadherin were lower than those of the other two groups. These data suggest that tumours in the Sh-ISG15 group have the characteristics of rapid formation, vigorous proliferation of blood vessels and easy metastasis. In the Exo-ISG15 group, the expression of CD31, Ki67 and Vimentin was lower than that in the control group, the expression of ESRP1 and E-cadherin was higher than that in the control group, and a large area of necrosis appeared in the tumour centre, indicating that tumour growth was inhibited and not prone to metastasis.

Both the in vitro and in vivo experiments described above showed that ISG15 was a tumour suppressor because it inhibited lung ADC proliferation and EMT progression.

### Activation of the IFN pathway and upregulation of ISG15 expression are closely correlated with ESRP1

After upregulating ESRP1 expression in A549 cells (LV-ESRP1), we used gene microarray to detect changes in gene expression. Microarray analysis demonstrated that 341 genes were upregulated and that 351 genes were downregulated (Fig. 3a). Among those genes, the IFN pathway-related genes were enriched (Fig. 3b). The downstream protein levels of the IFN pathway showed a significant increase, of which IFIT1 increased by 10.152 and ISG15 increased by 7.429 times (Fig. 3c). To verify the reliability of the microarray results, we selected three genes, and corresponding proteins (STAT1, STAT2 and ISG15) were estimated by RT-PCR and western blotting in A549 and H1299 cells (Fig. 3d, e). These results are in accordance with the results of the microarray analysis, indicating that ESRP1 overexpression can stimulate the IFN pathway and upregulate ISG15, a key downstream protein of the IFN pathway. We carried out IHC staining of ESRP1 and ISG15 in 56 lung ADC tissues, and the results showed there is a positive correlation between ISG15 and ESRP1 (*r* = 0.781, *P* < 0.001).

### ESRP1 regulates the transcription of ISG15 through CREB

**Fig. 4:**
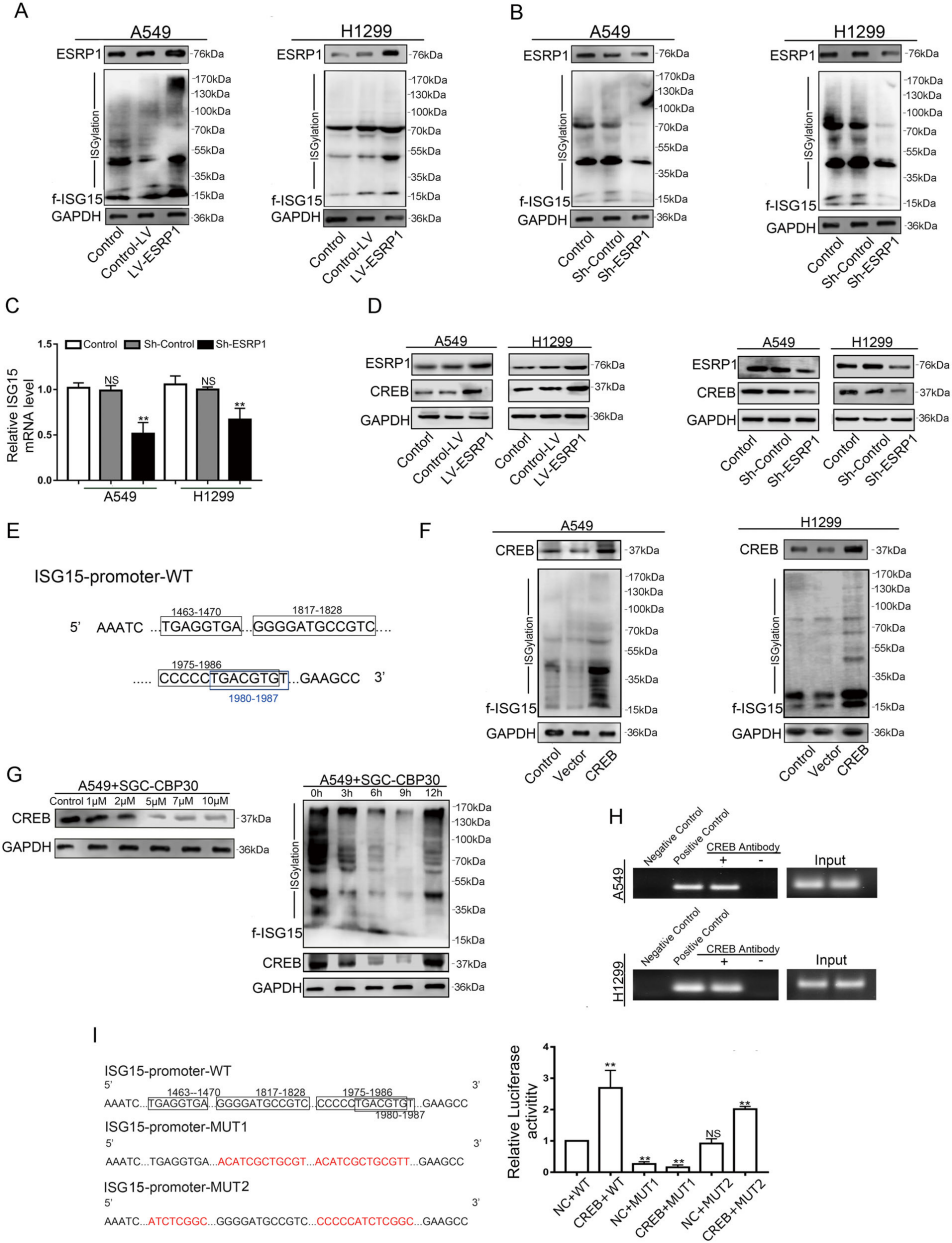
ESRP1 regulates the transcription of ISG15 through CREB. **a**, **b** Western blotting was performed to detect the expression level of ISG15 after up-regulated ESRP1 and knocked down ESRP1. **c** ISG15 mRNA levels of up-regulated ESRP1 and down-regulated ESRP1 in A549 and H1299 were detected by RT-qPCR. The data are shown as the mean ± SD. *P < 0.05, **P < 0.01. **d** Four sites that CREB may bind to in the ISG15 promoter. **e** The expression of CREB was assessed by Western blotting in A549 and H1299 cells that up-regulated ESRP1 and knocked down ESRP1. **f** Western blotting was used to detect changes in ISG15 after CREB transfection in A549 and H1299 cells. **g** Western blotting showed the effect of different concentrations of the CREB inhibitor SGC-CBP30 in the A549 cell line. ISG15 and CREB antibodies were used to detect changes in ISG15 after treatment with CREB inhibitor at different time points. **h** ChIP experiment between CREB and ISG15. PCR results showed that the CREB antibody group detected a signal. **i** The binding site of the ISG15 promoter region that may be associated with CREB, as well as the mutation site. The dual luciferase reporter system showed a decrease in relative fluorescence at MUT1. The data are shown as the mean ± SD. *P < 0.05, **P < 0.01.

To further study the relationship between ESRP1 and ISG15, we used Western blotting and RT-PCR to detect changes in protein and transcription levels of ISG15 in A549 and H1299 cells over- and underexpressing ESRP1. The Western blotting results demonstrated that ISG15 protein expression levels were upregulated following ESRP1 overexpression, while ESRP1 knockdown showed the opposite results (Fig. 4a, b). The RT-PCR results showed that similar changes occurred in the expression of ISG15 at the transcription level (Fig. 4c and Fig. 3e). By analysing the promoter region of ISG15, we found that its upstream promoter region has multiple transcription sites for CREB (Fig. 4d). We speculate that ESRP1 may regulate the transcription of ISG15 through CREB.

Initially, we evaluated CREB expression in the LV-ESRP1 group and Sh-ESRP1 group by Western blotting. The Western blotting results showed that the CREB level was increased in the LV-ESRP1 group and was decreased in the Sh-ESRP1 group (Fig. 4e). Subsequently, we performed transient transfection of CREB in A549 and H1299 cells. The Western blotting results showed that ISG15 significantly increased in the CREB-transfected group compared to the control group and the vector group (Fig. 4f). This result suggests that upregulation of CREB can promote the expression of ISG15. Then, we used the CREB-specific inhibitor SGC-CBP30 to observe changes in ISG15 expression after CREB inhibition. After treating A549 cells with 5 μM SCG-CBP30, the cells were harvested at 3, 6, 9 and 12 h. The Western blotting results showed that ISG15 first showed a downward trend and then switched to an upward trend over time. At 9 h, the ISG15 content was the lowest and gradually recovered. Regarding changes in CREB and ISG15, similar trends were generally observed (Fig.4g).

In addition, the ChIP results showed that CREB was able to bind to the promoter region of ISG15 (Fig. 4h). To further determine their specific binding sites, we performed a dual-luciferase reporter system assay. We mutated the binding sites to obtain MUT1 (mutated in the 1817-1828 and 1975-1987 regions) and MUT2 (mutated in the 1463-1470 and 1975-1987 regions) (Fig. 4i). The results showed that the MUT1 group had a significant decrease in relative luciferase activity. We speculated that the site of CREB binding to the ISG15 promoter region may be in the 1817 to 1828 range (Fig. 4i). The above results lead us to the conclusion that ESRP1 upregulates the expression of ISG15 through CREB. CREB can specifically bind to the 1817-1828 site of the ISG15 promoter region and enhance the transcription of ISG15.

### ESRP1 promotes ISG15 enrichment in the nucleus

**Fig. 5:**
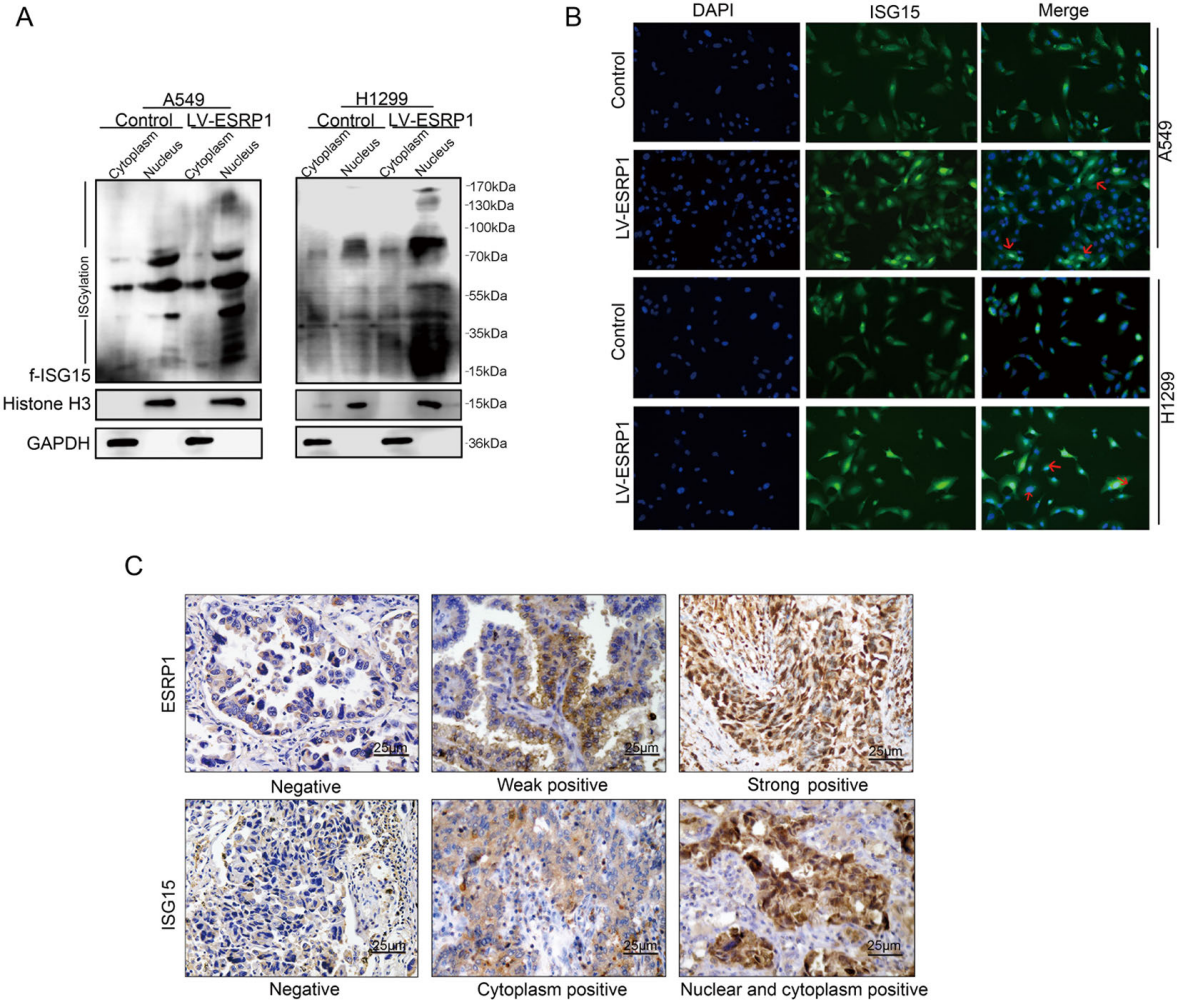
ESRP1 promotes ISG15 enrichment in the nucleus. **a** Cytoplasm and nucleus were separated and Western blotting was performed to detect the changes of ISG15 in different cell components after ESRP1 was up-regulated. **b** Cellular immunofluorescence staining was used to observe the expression level and localization of ISG15 after ESRP1 was up-regulated. **c** Immunohistochemical staining of ISG15 and ESRP1 was performed on serial sections of the same specimen. Magnification, ×400.

Since ISG15 subcellular localization leads to different prognoses, we observed the effect of ESRP1 overexpression on the localization of ISG15. We separated the nuclear and cytoplasmic fractions and used Western blotting to detect changes in different compartments of ISG15. The results showed that after upregulation of ESRP1, ISG15 expression in the nucleus increased significantly (Fig. 5a). Immunofluorescence staining showed that the nuclei in the LV-ESRP1 group became brighter than those in the control group (Fig. 5b). These results indicate that ESRP1 was able to upregulate ISG15 and that a higher expression of ISG15 was observed in the nucleus than in the cytoplasm. This phenomenon was also observed in lung ADC tissues. In lung ADC tissues weakly positive for ESRP1, ISG15 expression was also weakly positive, with almost no ISG15 expression in the nucleus (Fig. 5c). These results indicate that ESRP1 can upregulate ISG15 expression while enriching ISG15 in the nucleus. Combined with our existing conclusion that ESRP1 can inhibit the progression of lung ADC, this result explains why ISG15-positive patients tend to have better OS and RFS.

### ISG15 and ESRP1 combine to form ISGylation and keep ESRP1 from degradation

**Fig. 6:**
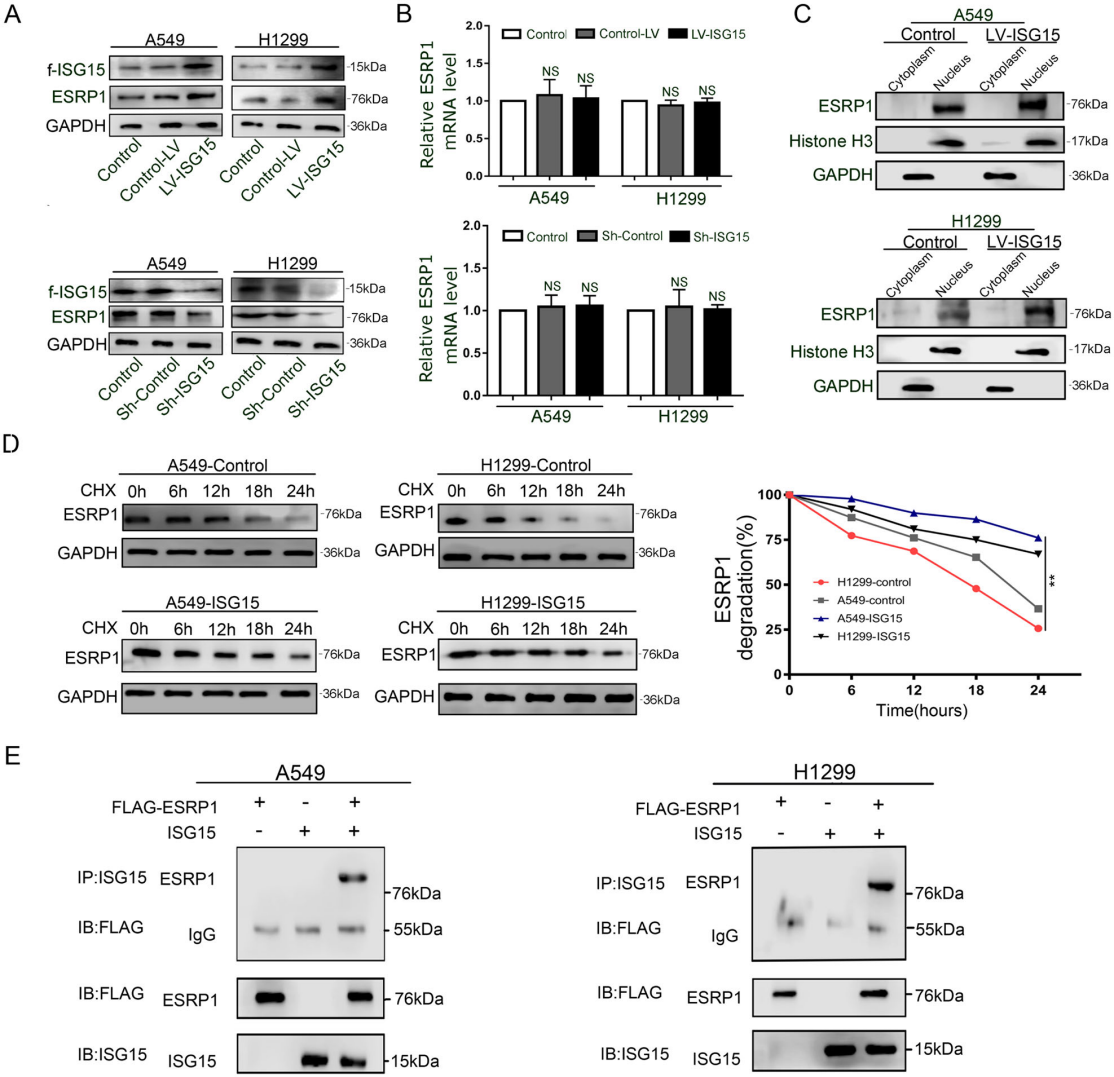
ISG15 and ESRP1 combine to form ISGylation, which prevents ESRP1 from degrading, and they synergistically inhibit EMT. **a**, **b** Western blot and RT-qPCR were conducted to examine the expression level of ESRP1 in cells that upregulated ISG15 and knocked down ISG15. The data are shown as the mean ± SD. * *P* < 0.05, ** *P* < 0.01. **c** The protein level of ISG15 in the cytoplasm and nucleus was detected by western blot, after up-regulation of ESRP1 treatment. ISG15 protein expression levels in the cytoplasm and nucleus were separately examined by performing Western blotting after up-regulated ESRP1. **d** Western blotting was performed to detect the expression level of ESRP1 at different times, after CHX treatment. The data were normalized for comparative purposes (right). The data are shown as the mean ± SD. *P < 0.05, **P < 0.01. **e** Co-IP assay was conducted with the anti-flag antibody in ESRP1-overexpressing and ISG15-overpression A549 and H1299 cells.

We evaluated the expression of ESRP1 in ISG15-overexpression (Ov-ISG15) and ISG15-knockdown (Sh-ISG15) A549 and H1299 cells. The results demonstrated that compared with those in the control group, the expression levels of ESRP1 protein were significantly increased in the Ov-ISG15 group and significantly decreased in the Sh-ISG15 group (Fig. 6a). Much to our surprise, the RT-qPCR results showed that the ESRP1 transcription levels in the Ov-ISG15 and Sh-ISG15 groups were almost unchanged compared to those of the control group (Fig. 6b). Obviously, changes in the levels of ESRP1 protein expression are not caused by altered gene transcription. Consistent with that in the control group, ESRP1 in the Ov-ISG15 group was localized in the nucleus, but its expression increased (Fig. 6c).As an important ubiquitin-like protein, ISG15 is likely to participate in the post-translational modification of ESRP1, making it more difficult to degrade ESRP1. Thus, we used CHX to assess the degradation rate of ESRP1 with both normal expression and overexpression of ISG15. In the control group, the level of ESRP1 began to show a significant decrease at 12 h, and 78% (A549) and 86% (H1299) of the proteins were degraded within 24 h. In the Ov-ISG15 group, the level of ESRP1 began to decrease at 18 h, and only 41% (A549) and 54% (H1299) of the proteins were degraded at 24 hours (Fig. 6d). The co-immunoprecipitation results indicate that there is a direct interaction between ESRP1 and ISG15. ISG15 promotes the ISGylation of ESRP1(Fig. 6e). These results indicate that ESRP1 is more stable and not easily degraded in the ISG15 upregulation group, which may be related to the occurrence of ISGylation between ISG15 and ESRP1.

### ISG15 and ESRP1 combine to suppress EMT

The above results demonstrate that ISG15 can be regulated by ESRP1 and enriched in the nucleus. ISG15 can perform ISGylation with ESRP1 and inhibit its degradation. We next set out to explore whether ISG15 and ESRP1 combine to suppress EMT. We generated stable ISG15-overexpression (ESRP1/ISG15 group) and ISG15-knockdown (ESRP1-ShISG15 group) based on ESRP1 overexpression (ESRP1 group) A549 and H1299 cells using lentiviral constructs.

**Fig. 7:**
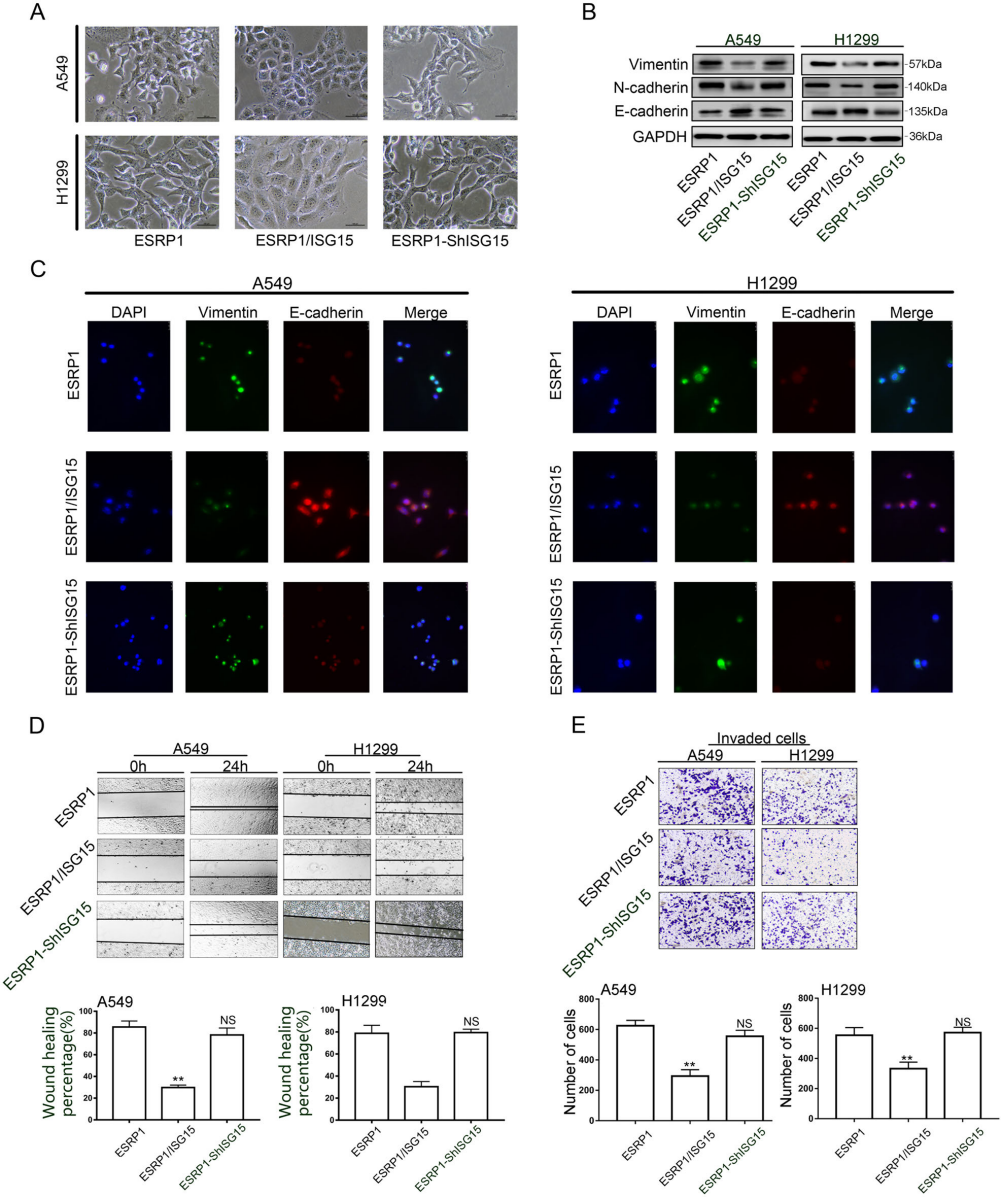
ISG15 and ESRP1 combine to suppress EMT. **a** On the basis of up-regulation of ESRP1, the morphological changes of A549 and H1299 cells after up-regulation or knockdown of ISG15. Magnification, ×200. **b** Western blotting was performed to detect the expression levels of EMT-related proteins, including E-cadherin, N-cadherin and Vimentin in three treatment groups. **c** Epithelial marker E-cadherin and mesenchymal marker Vimentin protein levels were determined by performing immunofluorescence staining of A549 and H1299 cells treated with the above three treatments. Magnification, ×200. **d**, **e** Transwell assay (magnification, ×200) and wound healing (magnification, ×100) were performed to detect the invasion and migration ability of cells in the three treatment groups. The data are shown as the mean ± SD. *P < 0.05, **P < 0.01.

Compared with the other two groups, the cells in the ESRP1 / ISG15 group are more closely aligned (Fig. 7a). The Western blotting results showed that compared with those in the control group, the levels of Vimentin and N-cadherin in the ESRP1/ISG15 group were significantly reduced, while that of E-cadherin was increased (Fig. 7b). However, compared with those in the control group, the changes in the levels of E-cadherin, N-cadherin and Vimentin in the ESRP1-Sh15 group were not statistically significant. The results of cellular immunofluorescence were the same as Western blotting. In the ESRP1 / ISG15 group, the expression of epithelioid marker E-cadherin was increased, and the mesenchymal marker Vimentin was decreased (Fig. 7c). The transwell assay and wound healing assay results also showed that ESRP1 combined with ISG15 could better inhibit the invasion and metastasis of lung adenocarcinoma cells (Fig. 7d, e).

## Discussion

In recent years, lung ADC has replaced squamous cell lung cancer as the most frequent cell type of NSCLC^21^. Lung ADC has a high rate of recurrence and distant metastasis. EMT remains an important factor that causes lung adenocarcinoma to tend to metastasize^22^.

ESRP1 acts as a regulator of alternative splicing events that help to suppress the EMT process in tumour progression. In previous articles, we have demonstrated the important function that ESRP1 plays in suppressing EMT^19^. In lung ADC tissues, ESRP1 overexpression was inversely related to the presence of metastases, tumour size, and clinical stage of lung ADC. Knocking down ESRP1 can also enhance the invasion and metastasis ability of A549 and H1299 cells. We have demonstrated the inhibitory effect of ESRP1 on EMT by two methods. One method shows that knockdown of ESRP1 can transform CD44 subtypes, that is, CD44v into CD44s^23^. The CD44s isoform promotes lung ADC cell invasion by regulating MMP-2 expression^19^. The other method shows that knocking down ESRP1 can convert FGFR2 IIIb to FGFR2 IIIc. FGF4 and FGF7 are ligands of FGFR2 IIIc and FGFR2 IIIb, respectively^4^. FGF4 instead of FGF7 altered cell morphology, promoted EMT-related protein expression, and enhanced the cell migration and invasion capabilities. In addition, FGF4 increased the expression of calcium-operated calcium storage (SOCE) and the calcium signal-related protein Orai1, which could also promote the EMT process in ADC cells. Using micro-array to detect A549 cells with ESRP1 overexpression, we found that the IFN pathway was significantly activated, and the level of its key downstream component ISG15 increased several times.

ISG15 was the first Ubl to be determined^24^. ISG15 can be strongly induced by type I IFN^25^. Many studies have confirmed that dysregulation of ISG15 and its conjugated compounds is found in many primary cancers, such as breast^10,26–28^, prostate^29^ and bladder cancers^30^, hepatocellular carcinoma (HCC) and oral squamous cell carcinoma^31^. In small cell and non-small cell type lung cancer patients, more than 70% of patients have a deletion in the chromosome 3p21 region, and UBE1L is located in this region, so it is suggested that ISG15 may be a tumour suppressor gene for lung cancer^32,33^.

By analysing ISG15 IHC staining in 153 patients with lung ADC, it was concluded that patients with high ISG15 expression usually have better OS and RFS. For the first time, the relationship between ISG15 nuclear positivity and clinical parameters was discussed separately. Patients with ISG15 nuclear-positive characteristics have the best prognosis and are not prone to long-term recurrence and metastasis. We first discovered that in patients with long-term recurrence and metastasis, the expression of ISG15 changed from high expression to low expression. This result strongly suggests that ISG15 is a good prognostic indicator in lung adenocarcinoma.

Studies have shown that camptothecin can induce ISG15 in fibrosarcoma cells^16^, and downregulation of ISG15 has been shown to reduce BC cell sensitivity to camptothecin^34^. In addition, it has been shown that the levels of ISG15 in tumours sensitive to irinotecan in gastric cancer patients are higher than those resistant to irinotecan in gastric cancer patients^35^. This observation indicates that antitumour drugs may exert a killing effect through ISG15. In our study, recombinant purified ISG15 not only inhibited cell proliferation but more importantly reduced the burden of xenografts in nude mice. In advanced high-grade serous ovarian cancer, recombinant purified ISG15 has also been shown to suppress tumour progression in tumour-bearing animals^36^.

We also clarified for the first time the regulatory mechanism of ESRP1 on ISG15; that is, ESRP1 upregulates the expression of ISG15 through CREB and enriches ISG15 in the nucleus. This mechanism explains why ISG15 nuclear-positive patients have better OS and RFS and are less likely to relapse and transfer. In addition, we found that ISG15 can promote the occurrence of ISGylation in ESRP1 and stabilize ESRP1. ISG15 and ESRP1 together inhibit EMT. Despite reports, KRAS-induced ISG15 and ISGylation in BC cells have the ability to inhibit the degradation of KRAS, thereby promoting BC cell proliferation, cell migration and EMT^10^. This high probability is caused by different target proteins of ISG15.

As a ubiquitin-like protein, ISG15 can not only bind to the target protein, but also act as an effector protein. The different roles of the two forms of ISG15 will be explored in subsequent studies. Similar to our results, many literatures have also indicate that ISG15 and the target proteins form ISGylation in the nucleus^37^, but the mechanism of how it enters the nucleus after being synthesized in the ribosomes of cytoplasm is still unclear, which is the direction of our further study. In addition, we observed that some cases of immune cells were positive for ISG15 IHC staining. ISG15 functions outside the cell in a free form, which is related to the production of cytokines and the activation of immune cells. Therefore, the effect of free ISG15 on immune cells in the tumour microenvironment will also be the focus of our further research. In summary, this article mainly describes the effect of ISG15 in lung adenocarcinoma and the interaction between ESRP1 and ISG15. For early-prone metastatic lung ADC, ISG15 can not only become a new prognostic indicator, but the combination of ISG15 and ESRP1 is also expected to become a new strategy for the treatment of ADC.

## Supplementary information

Editing certificate
