# Supplementary material for: ISG15 induces ESRP1 to inhibit lung adenocarcinoma progression
Source: Cell Death Dis. 2020 Jul 2;11(7):511. doi: 10.1038/s41419-020-2706-7 (PMC7343783; doi:10.1038/s41419-020-2706-7)
Supplement: Supplementary file 1 — Editing certificate [file 41419_2020_2706_MOESM1_ESM.pdf]

Editing Certificate

This document certifies that the manuscript

ISG15 interacts with ESRP1 to suppress EMT in lung adenocarcinoma

prepared by the authors

Qu tongyuan

was edited for proper English language, grammar, punctuation, spelling, and overall style by one or more of the highly qualified native English speaking editors at AJE.

This certificate was issued on **January 20, 2020** and may be verified on the AJE website using the verification code **F460-07E6-63D4-5272-23B9**.

Neither the research content nor the authors' intentions were altered in any way during the editing process. Documents receiving this certification should be English-ready for publication; however, the author has the ability to accept or reject our suggestions and changes. To verify the final AJE edited version, please visit our verification page at aje.com/certificate. If you have any questions or concerns about this edited document, please contact AJE at support@aje.com.

AJE provides a range of editing, translation, and manuscript services for researchers and publishers around the world. For more information about our company, services, and partner discounts, please visit aje.com.
